# Double, double toil and trouble: transforming growth factor beta (TGF-β) in HIV infection

**DOI:** 10.3389/fimmu.2025.1738092

**Published:** 2026-01-07

**Authors:** Jakob Harrison-Gleason, Kayla L. Yerlioglu, Ariel W. Halle, Judd F. Hultquist, Elena Martinelli

**Affiliations:** Department of Medicine, Division of Infectious Diseases, Feinberg School of Medicine, Northwestern University, Chicago, IL, United States

**Keywords:** chronic inflammation, fibrosis, HIV, latency, pathogenesis, SIV, TGF-β

## Abstract

Despite effective suppression of viral replication by antiretroviral therapy (ART), chronic HIV infection remains characterized by persistent low-level inflammation and progressive tissue damage, contributing to premature aging and an array of comorbidities including cardiovascular disease, HIV-associated neurocognitive disorders, liver disease, and fibrosis of multiple organs. Increased levels of transforming growth factor beta (TGF-β), characteristic of chronic HIV infection even in the context of ART, appear to be a common thread explaining these disparate comorbidities. As a pleiotropic cytokine with both immunosuppressive and pro-fibrotic properties, TGF-β exerts complex and sometimes paradoxical effects on the HIV lifecycle and pathogenesis. This review explores the multifaceted roles of TGF-β in HIV infection, with particular focus on three critical areas: immunosuppression, tissue fibrosis, and the regulation of viral latency. We discuss recent advancements in understanding the often-paradoxical role of TGF-β on HIV replication and latency dynamics, and how its different effects contribute to multiple mechanisms underlying HIV persistence, from inhibited immune responses and enhanced viral latency to impaired immune reconstitution. A more comprehensive understanding of the mechanisms by which TGF-β contributes to HIV persistence may illuminate novel therapeutic strategies targeting TGF-β signaling pathways for improved HIV treatment and progression toward functional cure.

## 1 Introduction

**Figure 1:**
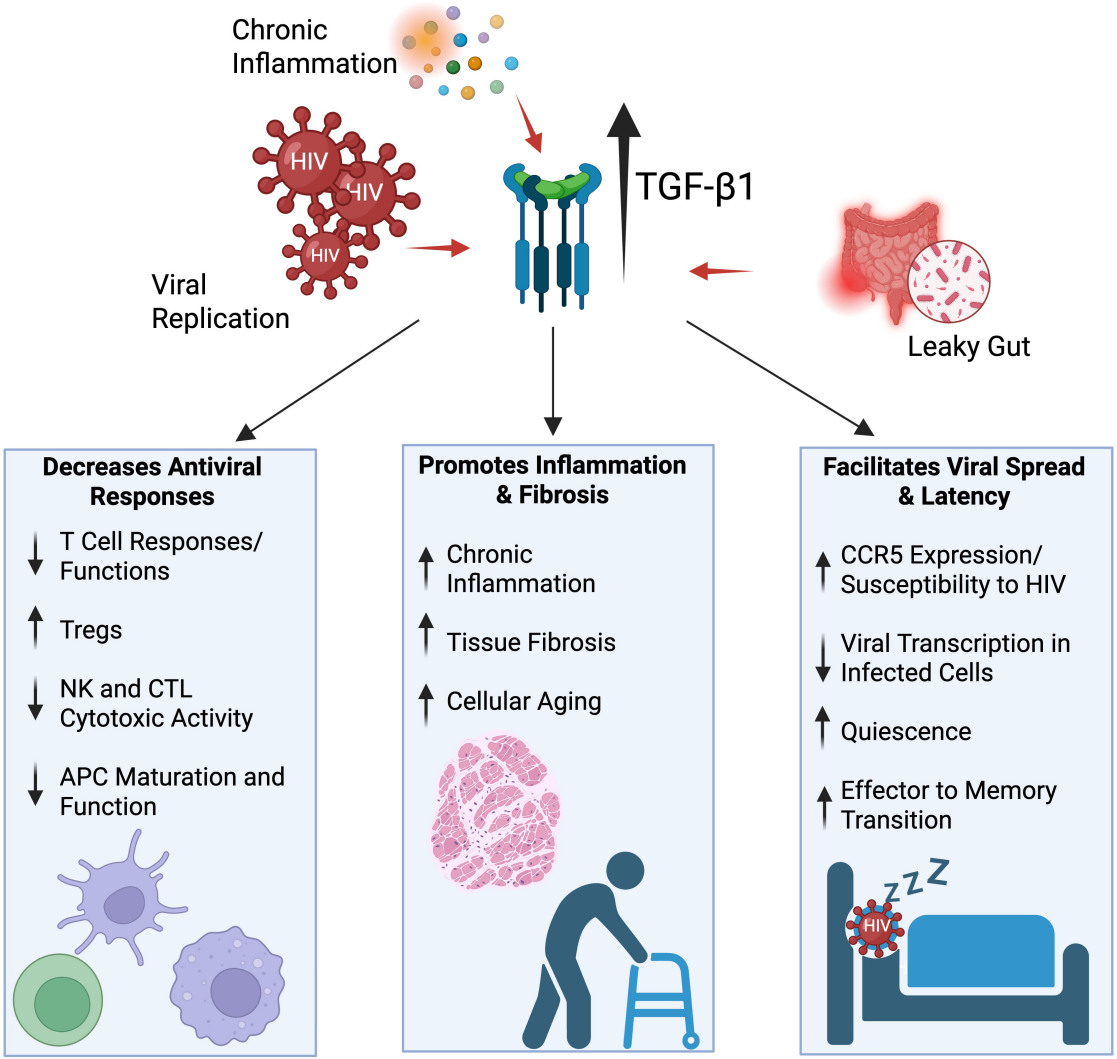
Graphical abstract. TGF-β inhibits immune responses to HIV, drives fibrosis and accelerated aging and increases both susceptibility to infection and HIV latency. Figure created with BioRender.com.

Human immunodeficiency virus (HIV) infection is characterized by progressive CD4^+^ T cell depletion, chronic immune activation, and the establishment of a proviral reservoir that persists despite effective antiretroviral therapy (ART) (1). While current ART effectively suppresses viral replication in people with HIV (PWH), it fails to eliminate the proviral reservoir, necessitating lifelong treatment to prevent disease progression (2, 3). Understanding the molecular mechanisms underlying HIV persistence requires examining key regulatory factors that influence viral latency and immune dysfunction. One such critical factor is transforming growth factor-beta (TGF-β). TGF-β is a pleiotropic cytokine that plays a crucial role in the regulation of immune responses, immune cell maturation, differentiation and activation, and the processes of tissue wound healing and fibrosis (4). There are three mammalian TGF-β isoforms (TGF-β1, TGF-β2, and TGF-β3) that signal through the same pair of transmembrane serine-threonine kinase receptors, TGF-β receptor I (TGFβRI) and II (TGFβRII). TGF-β1 is the dominant isoform in the immune cell compartment and plays the most important roles in immune response regulation (5). Therefore, we focus primarily on TGF-β1 in this review and ‘TGF-β’ will refer to TGF-β1 unless otherwise specified. Canonical TGF-β signaling involves TGF-β binding to TGFβRII, which recruits and phosphorylates TGFβRI (also known as ALK5) to activate its kinase domain. TGFβRI mediates phosphorylation of SMAD2/3, which subsequently form heteromeric complexes with SMAD4 and translocate to the nucleus to regulate target gene transcriptional programs (6). Additionally, TGF-β can signal through non-canonical, SMAD-independent pathways, including AKT (7), MAPK (8), and NF-kB (9). Through these canonical and non-canonical signaling networks, TGF-β exerts multifaceted effects on HIV pathogenesis by suppressing antiviral immune responses, promoting HIV-associated tissue fibrosis, and facilitating the establishment and maintenance of the persistent viral reservoir (**Figure 1**. Graphical abstract).

## 2 TGF-β levels are elevated in plasma and tissues of PWH

Extensive clinical and experimental evidence has demonstrated consistently elevated TGF-β levels across multiple biological compartments in PWH. TGF-β concentrations are significantly elevated in circulating plasma and serum from PWH compared to HIV-negative controls (10), regardless of ART treatment status or duration of viral suppression (11, 12). Even individuals who have achieved durable viral suppression on ART often maintain abnormally high TGF-β levels compared to people without HIV (PWoH) (7), indicating a fundamental alteration in immune homeostasis that occurs independently of active viral replication and instead likely results from chronic low-grade inflammation (13) and sustained immune activation (11, 14).

One factor likely contributing to this is the HIV-induced reduction in gut epithelium integrity, which results in microbial translocation (15) and activation of Toll-like receptor (TLR) signaling pathways that are triggered by sensing of bacterial byproducts like lipopolysaccharide (LPS) (16). Microbial sensing by gut macrophages leads to IL-1 family cytokines production and downstream secretion of inflammatory and regulatory cytokines, including TGF-β (17, 18). This establishes a mechanistic link between mucosal barrier dysfunction, heightened chronic inflammation, and increased profibrotic TGF-β signaling that persists despite effective ART. *Ex vivo* studies have provided additional evidence of TGF-β dysregulation at the cellular level, demonstrating that peripheral blood mononuclear cells (PBMCs) isolated from PWH spontaneously release significantly higher levels of TGF-β compared to those from PWoH (19).

Beyond systemic circulation, elevated TGF-β expression has been documented in multiple tissue types in PWH. In the brain, higher TGF-β levels have been observed in PWH (20, 21) as well as in SIV-positive non-human primates (NHPs) (22), with TGF-β levels directly correlating with the extent of viral infiltration in the central nervous system (CNS). Elevated TGF-β has also been reported in lymphoid tissues (23), where it stimulates fibrosis and impairs immune reconstitution (24) (see below), as well as in the kidneys (25), gut (26), and lungs (27). Overall, this suggests that heightened TGF-β expression is a pervasive feature of HIV disease progression regardless of the anatomical context, contributing to both sustained immunosuppression and end-organ damage.

Indeed, clinical studies have established significant correlations between elevated TGF-β levels and multiple indicators of HIV disease severity, including accelerated disease progression (28), lower CD4^+^ T cell counts (29), higher plasma viral loads (30), and increased systemic inflammation (11, 14). Collectively, these findings underscore TGF-β as a central player in HIV pathogenesis and highlight its potential utility as both a biomarker of HIV progression and as a therapeutic target (31).

## 3 TGF-β-mediated immunosuppression in HIV infection

**Figure 2:**
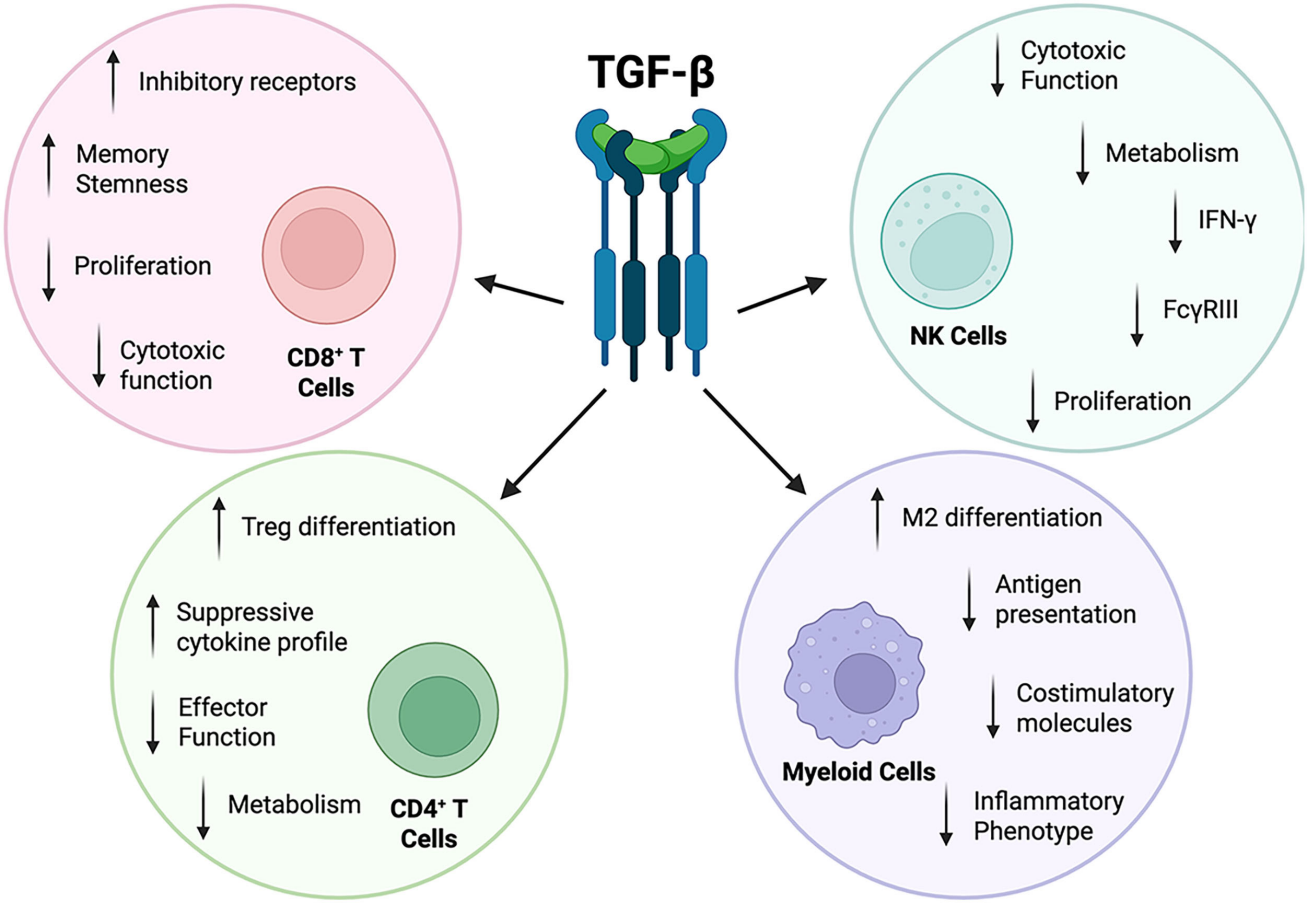
TGF-β inhibits immune responses to HIV. TGF-β exerts immunosuppressive effects on both the innate and adaptive immune cell compartments by inhibiting cytotoxicity, antigen presentation, and effector functions, while promoting immune tolerance. Figure created with BioRender.com.

TGF-β functions as a master regulator of immune homeostasis, orchestrating multifaceted immunosuppressive programs that impair antiviral responses in HIV infection (32, 33). Through coordinated effects on adaptive and innate immune compartments, TGF-β contributes to conditions that enable viral spread and persistence by suppressing T cell effector functions (34), compromising antigen presentation (35, 36), and promoting regulatory phenotypes across multiple cell lineages (37). These mechanisms, summarized below in the context of HIV, collectively weaken host antiviral immunity and sustain an immune environment in which infected cells are not efficiently cleared (**Figure 2****).**

### 3.1 T cell responses

TGF-β has a multi-faceted, negative impact on both CD4^+^ and CD8^+^ T cell proliferation and effector function, ultimately limiting clearance of infected cells (38). One primary mechanism by which it does this is through downregulation of IL-2 production, a cytokine essential for both CD4^+^ and CD8^+^ T cell proliferation and activation (39, 40). Combined with virus-driven CD4^+^ T cell loss (41), this TGF-β-driven reduced proliferative capacity and reduced responsiveness to co-stimulation (42) weakens the immune system’s ability to clear infected cells and control viral spread. In support of this, PBMCs from PWH exhibit defective proliferation in response to recall antigens, which can be partially restored by neutralizing TGF-β with monoclonal antibodies (31). Elevated TGF-β also promotes CD4^+^ T cell differentiation into regulatory T cells (Tregs) (43), which release additional TGF-β and suppressive cytokines like IL-10 (44), thereby amplifying immunosuppression. The expansion of Tregs in HIV has been directly linked to increased TGF-β production, establishing a positive feedback loop that suppresses effector T cell function (45, 46). Moreover, studies in SIV-infected ART-treated macaques have shown that Treg/Th17 balance is altered in gut-associated lymphoid tissues (GALT): higher Treg frequencies persist together with elevated TGF-β and IDO expression in mesenteric lymph nodes (MLN), even under therapy (47), suggesting that TGF-β–driven immunoregulation remains active in mucosal and gut-associated compartments despite viral suppression.

Beyond its effects on proliferation and Treg induction, TGF-β signaling also intersects with inhibitory checkpoint pathways and T follicular helper (Tfh) differentiation programs. TGF-β has been shown to increase PD-1 expression on T cells in chronic infection, reinforcing an exhausted, hyporesponsive phenotype (48, 49). PD-1^hi^ Tfh cells, in turn, represent a major reservoir for HIV in lymphoid tissues and B cell follicles (50, 51). Consistent with this, recent work demonstrates that TGF-β promotes Tfh differentiation and humoral responses at least in part by inhibiting expression of the chromatin organizer SATB1 (52). Together, these findings suggest that higher TGF-β not only dampens antiviral effector responses but also favors the generation and maintenance of Tfh cells that function as key tissue reservoir.

Importantly, TGF-β has been shown to directly inhibit terminal differentiation and cytotoxic activity of antiviral CD4^+^ T cells during chronic viral infections, supporting an undifferentiated memory phenotype through decreased inhibition of eomesodermin (53). Beyond the direct suppression of effector functions, emerging evidence indicates that TGF-β impacts CD4^+^ T cell metabolism, reducing mitochondrial respiration and glycolytic capacity (54). These metabolic changes are known to restrict T cell activation and cytokine production, although this mechanism remains relatively underexplored in the context of HIV infection.

In CD8^+^ T cells, TGF-β has been shown to suppress both proliferative capacity and cytotoxic activity (55). TGF-β directly reduces CD8^+^ T cell proliferation by inducing cyclin-dependent kinase inhibitors such as p21 (56) and p15 (57) to inhibit cell-cycle progression. In addition, TGF-β interferes with IL-15 signaling, which is essential for long-term CD8^+^ T cell survival and memory formation (58). Beyond reducing proliferative capacity, TGF-β also suppresses cytotoxic function by downregulating the expression of key effector proteins –including perforin, granzyme A and B, Fas ligand, and interferon-γ (31, 59). Neutralization of TGF-β *in vitro* has been shown to restore perforin expression in rectal CD8^+^ T cells, providing evidence that TGF-β directly limits effector function in HIV-targeted tissues (60). Importantly, in SIV-infected macaques, elevated TGF-β (and IDO) expression within intestinal lymphoid tissues was associated with increased death of effector/memory CD8^+^ T cells via a Bax/Bak/Puma–dependent apoptotic pathway; conversely, *in vitro* blockade of TGF-β enhanced T cell proliferation and reduced CD8^+^ T-cell death (61). This evidence suggests that TGF-β–mediated depletion of cytotoxic CD8^+^ T cells in mucosal/lymphoid reservoirs can contribute to inefficient viral control during chronic infection.

*In vivo*, TGF-β blockade in SIV infected, ART-treated macaques enhanced SIV-specific CD8^+^ and CD4^+^ T cells responses providing direct evidence that inhibiting TGF-β can boost antiviral immunity (62, 63). Furthermore, FoxP3^+^ CD8^+^ Treg cells in PWH and SIV-infected NHPs have been found to express TGF-β along with inhibitory receptors, such as PD-1 and CTLA-4. These populations have reduced cytokine secretion and proliferative capacity, reflective of an exhausted phenotype (64). This occurs in part through lower expression of the transcription factor T-bet, a critical regulator of cytotoxic gene programs (65). On the other hand, TGF-β appears to be critical to the maintenance of stemness in pre-exhausted Tpex cells and to form long-term CD8^+^ T cell memory in chronic LCMV infection (49). A comprehensive of the impact of TGF-β on T cells can has been published by Chen W et al. in 2023 (5).

### 3.2 Myeloid cells

In myeloid lineage cells, particularly macrophages and dendritic cells, TGF-β significantly impairs antigen-presentation and inflammatory functions, creating additional barriers to effective antiviral immunity. TGF-β has been shown to inhibit Class II Transactivator (CIITA), the master regulator of MHC class II gene expression, thereby decreasing cell surface levels of MHC II and reducing antigen presentation capacity (66, 67). As a result, TGF-β inhibits the ability of macrophages and dendritic cells to effectively engage T cell receptors (TCRs) and stimulate adaptive responses. TGF-β also downregulates co-stimulatory molecules, such as CD80 and CD86, limiting the secondary signals required for full T cell activation (68). In the context of HIV infection, exposure to elevated TGF-β was shown to alter dendritic cell maturation, diminishing IL-12 secretion and skewing differentiation toward a tolerogenic phenotype that compromises the initiation of Th1-driven antiviral immunity (68, 69). Similarly, studies of monocyte/macrophage polarization show that TGF-β, alone or in combination with IL-10 and IL-4, drives cells toward an immunosuppressive “M2-like” phenotype characterized by reduced antigen-presentation capacity and enhanced tissue-repair function (70). A more recent report suggests that TGF-β leads to metabolic reprogramming that ultimately induces a distinct macrophage phenotype characterized by elevated expression of genes associated with a pro-repair phenotype (such as tissue remodeling, vasculature development, negative regulation of TNF production) and decreased expression of genes associated with inflammation (71). Of note, in SIV infected macaques on ART, blocking TGF-β signaling led to a more tolerogenic phenotype in lymph node macrophages (62). Collectively, these findings demonstrate that TGF-β not only directly suppresses lymphocyte effector functions but also fundamentally alters the activation state and functional capacity of antigen-presenting cells, thereby compromising immune coordination and antiviral responses in PWH.

### 3.3 Natural killer cells

Natural killer (NK) cells play a crucial role in controlling HIV-1 replication during acute and chronic infection through both direct cytotoxic activity and immunoregulatory functions (72–74). NK cell function has been shown to modulate virologic control in HIV-1 elite controllers, who maintain viral suppression in the absence of ART, and in long-term non-progressors, who control HIV-1 disease progression in the absence of ART (75). In NHP models, a subset of NKG2a/c^low^ CD16^+^ cytotoxic NK cells in the lymph nodes has been associated with virologic control in non-pathogenic SIV infection and in pathogenic SIV infection upon ART interruption (76). TGF-β restricts NK cell number and effector function in chronic viral infection and inhibits NK cell cytotoxicity and IFN-γ production (77). Notably, TGF-β specifically inhibits CD16 (FcγRIIIa)-driven IFN-γ production and NK cell antibody-dependent cellular cytotoxicity (ADCC) capability via SMAD3 signaling (78), with blockade of TGF-β being sufficient to upregulate CD16 expression on NK cells *in vivo* (62). This is particularly relevant in the context of current therapeutic and curative strategies involving anti-HIV broadly neutralizing antibodies (bNAbs), which leverage CD16 signaling to engage in NK cell-mediated ADCC (79, 80). Beyond these direct functional effects, TGF-β also profoundly impacts NK cell metabolism by suppressing glycolysis and oxidative phosphorylation, thereby limiting the bioenergetic capacity required for optimal NK cell effector responses (81, 82).

In addition to classical NK cells, TGF-β also modulates other innate lymphocyte subsets. In pathogenic SIVmac infection, the acute phase is marked by early elevations of both TGF-β and IL-18 in intestinal tissues, which drive the emergence of highly inflammatory IL-17–expressing NKT^+^ cells (83). These IL-17^+^ innate-like lymphocytes are absent in non-pathogenic SIVagm infection (83), which also does not lead to an increase in TGF-β/IL-18–associated pathways (84), supporting a link between elevated TGF-β and pathogenic outcomes.

Collectively, TGF-β-mediated suppression of T cell responses and cytotoxic activity, impaired antigen presentation by myeloid cells, and compromised NK cell effector functions establish TGF-β as a critical mediator of HIV-driven immune dysfunction and viral persistence (**Figure 2**). Evidence from clinical studies with PWH and NHP studies indicate that robust CD8^+^ T cell and NK cell responses can synergistically eliminate infected cells, contribute to virologic control on ART, and delay viral rebound following ART interruption (85–87). Hence, strategies to counteract TGF-β-mediated immunosuppression may be key to restoring immunological function and achieving host-mediated virologic control in the absence of ART.

## 4 TGF-β As primary driver of fibrosis and accelerated aging in HIV

### 4.1 TGF-β and HIV-driven fibrosis

One of the clinical hallmarks of long-term HIV infection is the development of fibrosis throughout multiple organ systems, with lymphoid tissues being particularly affected (31). In both untreated and treated HIV infection, extensive collagen deposition in lymphoid tissues drives profound pathological changes, leading to complete loss of tissue architecture, paracortical T cell zone damage and depletion of CD4^+^ T cells (88, 89). Even in PWH who have achieved virological suppression on ART, tissue fibrosis progresses through persistent pro-fibrotic signaling and chronic low-grade inflammation (90). The expansion of fibrotic regions impairs immune reconstitution and tissue repair mechanisms, thereby compounding HIV-associated tissue damage and pathogenesis while contributing to viral persistence (91).

**Figure 3:**
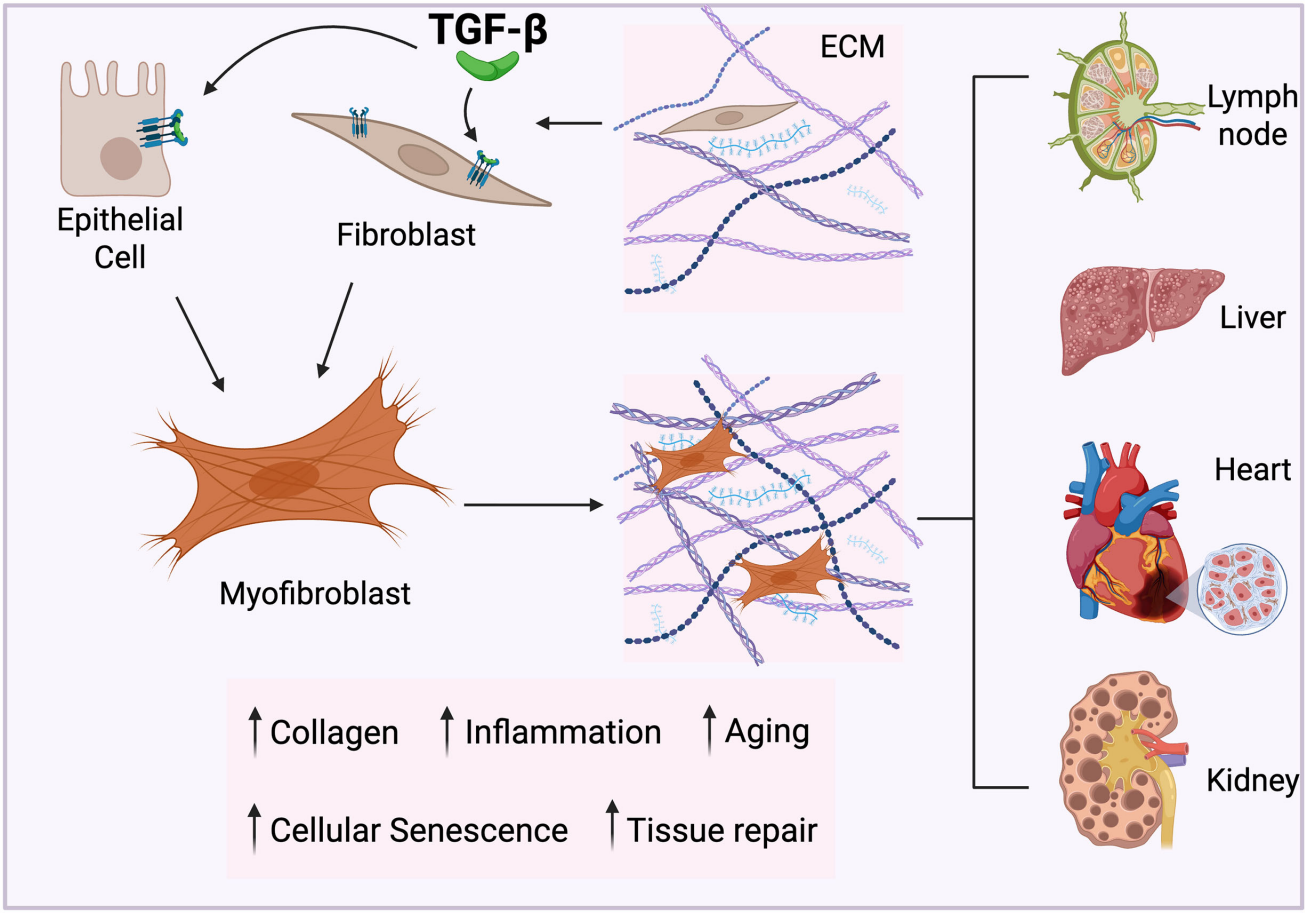
TGF-β drives fibrosis and accelerated tissue aging in PWH. Increased TGF-β levels in PWH promote the differentiation of fibroblasts and epithelial cells into myofibroblasts, which secrete excessive amounts of collagen relative to cells present in healthy tissue. TGF-β also limits the degradation of the extracellular matrix (ECM) by downregulating matrix metalloproteinase expression, resulting in increased ECM deposition and a shift towards fibrotic tissue architecture. This in turn disrupts stem cell niches and limits regenerative capacity, impairing organ function and driving the onset of premature aging across diverse organ systems. Figure created with BioRender.com.

Fibrosis in the context of HIV infection is strongly driven by TGF-β, which promotes the excess deposition and limited turnover of extracellular matrix (ECM) proteins that form the basis of fibrotic tissue (92, 93). In healthy individuals, the transient deposition of ECM proteins facilitates wound healing with matrix metalloproteinases (MMPs) promoting ECM turnover to prevent pathological scarring. However, when TGF-β remains chronically elevated during long-term HIV infection, ECM proteins can accumulate pathologically and lead to the development of fibrotic tissue (**Figure 3**). TGF-β promotes the differentiation of epithelial cells and fibroblasts into myofibroblasts (92, 93), which are markedly more active in producing ECM proteins, such as collagen, in comparison to resident cells in healthy tissues (94). Furthermore, TGF-β also inhibits ECM degradation by downregulation of MMP expression, which impairs ECM clearance and amplifies fibrotic remodeling (88). Pharmacologic targeting of the TGF-β pathway, such as with anti-fibrotic drug pirfenidone, significantly reduced myofibroblast-driven ECM production, reinforcing TGF-β’s central role in HIV-associated lymphoid fibrosis (24). Importantly, treatment of rhesus macaques with pirfenidone at the time of SIV infection prevented lymphoid tissue fibrosis *in vivo* and was associated with preservation of CD4^+^ T cells in both lymph nodes and blood, while administration post-infection was less effective (95). In these studies, interference with TGF-β signaling by pirfenidone was responsible for the effect, confirming TGF-β as a target for prevention or reversal of lymphoid fibrosis and improved immune reconstitution in HIV (95).

While lymphoid tissue fibrosis is a hallmark of long-term HIV infection (31), TGF-β is also strongly implicated in fibrogenesis in several non-lymphoid tissues (**Figure 3**). In the liver, elevated TGF-β acts synergistically with IFN-γ to enhance inflammatory and fibrotic pathways, contributing to non-alcoholic steatohepatitis (NASH) and metabolic dysfunction-associated steatotic liver disease (MASLD) in both humanized mouse models and in PWH (96, 97). Notably, MASLD in PWH has been reported to exhibit more advanced stages of fibrosis despite lower inflammatory activity compared to uninfected controls with MASLD, suggesting an outsized role for TGF-β in driving disease in PWH (98). Recent studies in SIV-infected macaques further support a central role for TGF-β in hepatic inflammation and fibrogenesis. These analyses revealed that, in addition to innate immune populations (99), hepatic CD4^+^ T cells are directly infected during SIV infection and exhibit elevated TGF-β mRNA expression (100). The presence of TGFB1-expressing infected CD4^+^ T cells within the liver microenvironment provides an additional localized source of TGF-β, potentially amplifying pro-fibrotic signaling and contributing to NASH-like pathology in chronic infection. Clinical trials of hydronidone, a modified pirfenidone derivative designed to more specifically target hepatic fibrosis via inhibition of TGF-β signaling, have shown significant benefit in Hepatitis B-driven liver fibrosis (101), suggesting that similar pharmacological approaches may improve liver fibrosis in the context of HIV.

Elevated TGF-β levels have also been associated with HIV-linked cardiac fibrosis and increased cardiovascular risk in PWH (102, 103). Cardiac magnetic resonance imaging (MRI) studies have demonstrated that myocardial fibrosis is three to four times more prevalent in PWH compared to PWoH (104). Pirfenidone has demonstrated efficacy in reducing myocardial fibrosis in heart failure patients, as evidenced by decreased myocardial extracellular volume after 52 weeks of treatment (105). However, because TGF-β also contributes to tissue repair following myocardial infarction, larger clinical trials are required to determine whether TGF-β inhibition represents a safe and effective strategy for managing HIV-associated cardiac fibrosis.

Additionally, TGF-β has been linked to worsened renal disease and chronic nephropathy in PWH through progressive fibrotic accumulation (25, 106). Unlike hepatic and myocardial fibrosis, however, clinical strategies targeting TGF-β in renal disease have been largely unsuccessful. Clinical trials with LY2382770, a TGF-β-specific monoclonal antibody, were terminated early due to lack of efficacy in improving diabetes-associated nephropathy (107). Pirfenidone demonstrated modest improvement in glomerular filtration rate, but these studies were limited by small sample size and high dropout rates (108). Clinical studies on the therapeutic targeting of TGF-β in the context of renal disease have not focused on PWH as a treatment group, and further large-scale investigations are required to determine whether TGF-β inhibition has therapeutic potential in HIV-associated nephropathy.

### 4.2 TGF-β and accelerated aging

Beyond its role as a driver of tissue fibrosis, a process tightly linked to the development of aging-related disorders (109), TGF-β contributes in several other ways to the broader processes of immune and systemic aging in PWH. Long-term HIV infection, even in the context of ART-mediated viral suppression, is associated with the premature onset of age-related comorbidities relative to age-matched HIV-negative controls, including cardiovascular disease, renal disease, neurocognitive decline, and metabolic multimorbidity (110–112). The mechanisms driving this accelerated aging phenotype among PWH appear to be multifactorial, but TGF-β-mediated fibrosis, chronic inflammation, dysregulated cytokine signaling (as evidenced by elevated IL-6, TNF-α, and sCD14 levels (113)), and cellular senescence (88, 114) all appear to be central contributors. Persistent TGF-β signaling promotes several of these factors that accelerate immunologic and systemic aging (89, 115, 116), providing a mechanistic link between chronic inflammation and accelerated aging observed in PWH.

TGF-β-driven fibrosis mirrors several core mechanisms of organ aging. The resulting ECM stiffening and architectural distortion impair tissue elasticity, disrupt stem-cell niches, and limit regenerative capacity, mechanisms that align closely with hallmarks of aging such as stem-cell exhaustion, cellular senescence, and impaired immune regeneration. In PWH, these fibrosis-aging dynamics emerge early and persist despite ART. For example, fibro-collagenous remodeling in lymph nodes and gut-associated lymphoid tissue has been shown to restrict access to key T cell survival signals (*e.g.*, IL-7), compromise CD4^+^ T-cell homeostasis, and interfere with immune reconstitution, functionally “aging” the immune microenvironment even in virologically suppressed individuals (115). Furthermore, stiffening of the ECM has been shown to induce senescence in alveolar epithelial cells in pulmonary fibrosis models (117) and mesenchymal stromal cells (stem/progenitor cell populations) in PWH show functional deficits in immunological non-responders (PWH who achieve viral suppression with ART, but do not fully restore their CD4^+^ T cell counts) (118). Thus, fibrosis reflects a convergent pathway through which TGF-β-based signaling reshapes tissue mechanics and repair programs, linking physiologic aging and HIV to a shared framework of premature multimorbidity in PWH.

Beyond fibrosis, TGF-β may also directly contribute to the epigenetic and metabolic reprogramming of immune and stem cell compartments in PWH. CD4^+^ T cells from PWH on suppressive ART exhibit transcriptional and metabolic signatures resembling those of older uninfected individuals, including elevated exhaustion marker expression, reduced mitochondrial function, and impaired proliferative capacity (119). These dysfunctions may be amplified in TGF-β-rich environments, potentially promoting effector-to-regulatory shifts and activating SMAD-mediated transcriptional programs that reinforce cellular senescence.

TGF-β can also influence the stem and progenitor cell compartments altering differentiation potential and limiting self-renewal. Hematopoietic stem cells (HSCs) in aging individuals and PWH often exhibit skewed differentiation potential (with a notable bias towards myeloid cells), reduced self-renewal, and functional exhaustion—phenotypes linked to increased TGF-β signaling in the bone marrow microenvironment (120, 121). Likewise, in pigtailed macaques with asymptomatic SIV infection, elevated plasma TGF-β levels were inversely correlated with hepatic thrombopoietin (THPO) transcription and bone marrow megakaryocyte density, suggesting inhibitory effects on megakaryopoiesis and broader hematopoietic dysfunction (122).

Together, these findings position TGF-β as a potential central regulator of premature aging in PWH, affecting immune function, metabolic balance, epigenetic programming, and stem-cell maintenance. However, additional research is needed to determine whether therapeutic modulation of TGF-β can safely and effectively alleviate HIV-driven accelerated aging, particularly within dysfunctional and fibrotic tissues.

## 5 TGF-β’s role in HIV replication and latency

### 5.1 TGF-β in HIV replication

**Figure 4:**
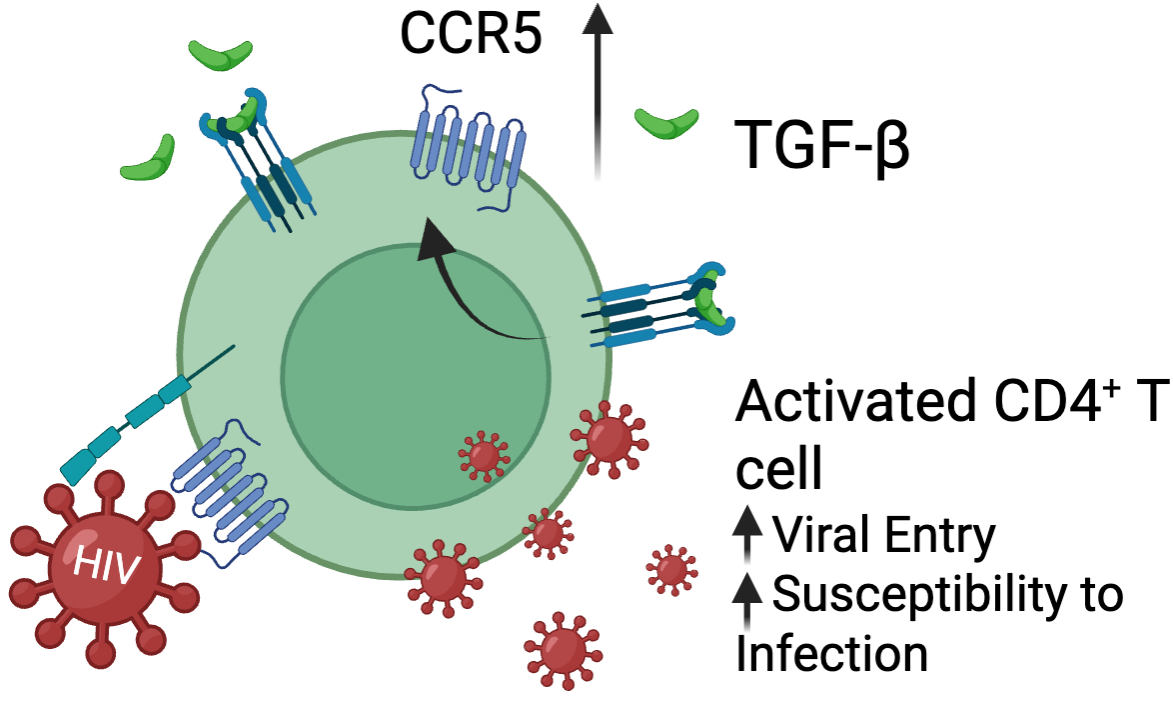
TGF-β upregulates CCR5 on T cells. TGF-β increases the expression of HIV-1 coreceptor CCR5 on activated memory CD4^+^ T cells facilitating new infection. Figure created with BioRender.com.

TGF-β signaling causes multiple downstream effects that can either enhance or inhibit HIV replication depending on the cell type and context. During the acute phase of HIV infection, activated CD4^+^ T cells that express high levels of the CCR5 co-receptor are preferentially infected (1, 123, 124). The level of CCR5 on the cell surface is strongly influenced by cytokines and T cell activation signals. For example, IL-6 and several Th1-type, pro-inflammatory cytokines (such as TNF-α, IL-2, and IL-12) have been reported to upregulate CCR5, whereas other T cell stimulation and other cytokines have been reported to downregulate it in a cell type dependent manner (125–127). TGF-β has been shown to increase CCR5 expression and to promote infection of both resting and activated memory CD4^+^ T cells *in vitro* (**Figure 4**), suggesting that TGF-β can enhance CCR5-tropic virus spread under certain conditions (128, 129). This has been similarly observed in myeloid cells, with one report showing that TGF-β increases viral replication in monocyte-derived macrophages (130).

By contrast, several other reports have shown that TGF-β suppresses HIV expression and spread in cells that are already chronically infected—for example, potent inhibition of virus production was observed in the promonocytic U1 cell line after TGF-β treatment (131–133). This inhibitory effect has been linked to direct modulation of HIV LTR activity by the TGF-β signaling cascade [for example, by regulation of non-canonical NF-κB (134) or BLIMP-1 (135)]. A more detailed discussion of the impact of TGF-β on HIV transcription is included in the next section. Beyond these direct transcriptional effects, broader phenotypic changes in TGF-β-treated HIV-1-target cells may likewise alter viral restriction factors and/or susceptibility to infection [*i.e.*, by impacting cell differentiation (43, 136, 137), triggering changes in cell adhesion and trafficking (137, 138), or via metabolic reprogramming (54, 139)], but much more work in this area is still required.

These apparently discordant observations can be reconciled with some attention to experimental context: studies that report TGF-β–mediated increases in viral replication measured infection after exposing uninfected primary cells to virus in the presence of TGF-β, whereas the reports of suppression measured virus production from cell lines or primary cells already harboring integrated provirus. Taken together, these data are consistent with TGF-β playing a multifaceted, context-dependent role in HIV infection. During active replication (*i.e.*, during acute infection or in the absence of antiretroviral therapy), TGF-β may increase susceptibility to infection and facilitate viral spread by inducing the expression of CCR5 (**Figure 4**), whereas in already infected cells, TGF-β signaling may inhibit viral gene expression and contribute to latency.

### 5.2 TGF-β as driver of HIV-1 latency

HIV persists in long-lived latent reservoirs composed primarily of quiescent CD4^+^ T cells that harbor integrated, intact DNA proviruses, but that do not actively produce replication-competent virions due to transcriptional or post-transcriptional blocks to viral gene expression (140, 141). Given that immune recognition largely depends on viral antigen expression, these latently infected cells can evade immune clearance and persist even under long-term suppressive ART (142, 143). This persistent viral reservoir represents the main barrier to an HIV cure (144, 145).

Initial studies of HIV latency were performed in immortalized cell line models. T cell line models including CEM-derived (*i.e.*, 8E5, ACH-2) and Jurkat-derived (*i.e.*, J-Lat, J1.1, Jurkat E4) lines demonstrated that cells with an integrated provirus can remain transcriptionally silent without producing infectious virions (146). These cell lines differ in their baseline levels of viral gene expression, in the site(s) of proviral integration, and in viral genotype, all of which can influence maintenance of the latent state and reactivation in response to stimulation (146). A large number of blocks to proviral gene expression have been described in these models, including epigenetic, transcriptional, and post-transcriptional blocks. Some of these blocks can be overcome by exposure to latency-reversing agents (LRAs) that stimulate viral gene expression through a variety of mechanisms. Well-characterized LRAs include: PKC agonists such as PMA (phorbol 12-myristate 13-acetate) (147), canonical NF-kB agonists such as TNF-α (Tumor necrosis factor alpha) (148), non-canonical NF-kB agonists such as AZD5582 (149), histone deacetylases (HDAC) inhibitors such as vorinostat (150), BET bromodomain inhibitors such as JQ1 (151), etc. Analogous models have also been established in myeloid lineages. The U1 cell line (derived from U937 promonocytes) and the OM10.1 cell line (derived from HL-60 promyelocytes) similarly maintain integrated, but transcriptionally inhibited, proviruses, which can be reactivated by cellular stimulation or cytokine exposure (152).

Early work with the U1 cell line showed that PMA-induced viral reactivation could be dampened by co-treatment with TGF-β (132). Interestingly, the TGF-β driven decrease in reactivation was not observed when TNF-α was used as the LRA in the same study, even though both PMA and TNF-α are thought to act through activation of the NF-kB pathway (153, 154). Similar results have been reported in the ACH-2 T-cell model, with TGF-β dampening viral reactivation upon PMA treatment (133). The determinants underlying the repressive effect TGF-β has on some LRAs in some cell line models of latency remain to be fully described. While the mechanistic basis for TGF-β’s inhibitory effects on reactivation requires further exploration, these findings suggest that TGF-β’s latency-enforcing properties may extend across diverse cell types that compose the reservoir. Comprehensive investigations examining TGF-β effects in diverse latency models, encompassing both myeloid-derived and T-cell-derived systems, will be essential to fully understand how this cytokine influences HIV latency dynamics in different cellular contexts.

TGF-β’s involvement in HIV latency has also been examined in primary cells and cells from PWH, which offer more physiologically relevant insights than immortalized cell lines. Primary cell latency model development has concentrated predominantly on memory CD4^+^ T cell populations, as memory cells exist in a quiescent state and constitute the majority of the HIV reservoir *in vivo* (155–157). The first documented example of TGF-β contributing to HIV latency in primary T cells occurred during early efforts to establish a T_cm_ differentiation protocol for latency induction (158, 159). In these early studies, researchers aimed to differentiate T_cm_ cells from naïve CD4^+^ T cells by activating them with αCD3/αCD28 in presence of αIL-4, αIL-12, and TGF-β. Following HIV infection, these differentiated cells maintained viral latency and could be reactivated upon stimulation with αCD3/αCD28 and IL-2. While TGF-β’s latency-promoting properties were not the intended focus of these experiments, this work provided the first evidence that TGF-β could facilitate HIV latency establishment in primary T cells (158, 159).

This model of latency generation was subsequently refined by a different group, who developed the QUECEL (quiescent effector cell latency) model to generate well-defined, quiescent memory T cells through controlled differentiation and polarization (160). In this approach, naïve CD4^+^ T cells are first activated with αCD3/αCD28 in the presence of specific polarization cytokine cocktails to produce Th1, Th2, Th17, and Treg effector populations. These expanded effector cells are then infected with HIV-1 and driven into a memory-like latent state using a combination of TGF-β, IL-8, and IL-10. Although IL-8 and IL-10 alone were able to induce partial latency, the inclusion of TGF-β was required for full latency establishment in this model (160), underscoring its critical role in promoting the transition from active effector phenotypes to resting, memory-like states across multiple T cell subsets. Notably, the transcriptional and reactivation profiles of cells derived from the QUECEL model closely mirror those of *ex vivo* PBMCs isolated from PWH on long-term ART, highlighting its physiological relevance as a primary cell model for studying HIV latency.

**Figure 5:**
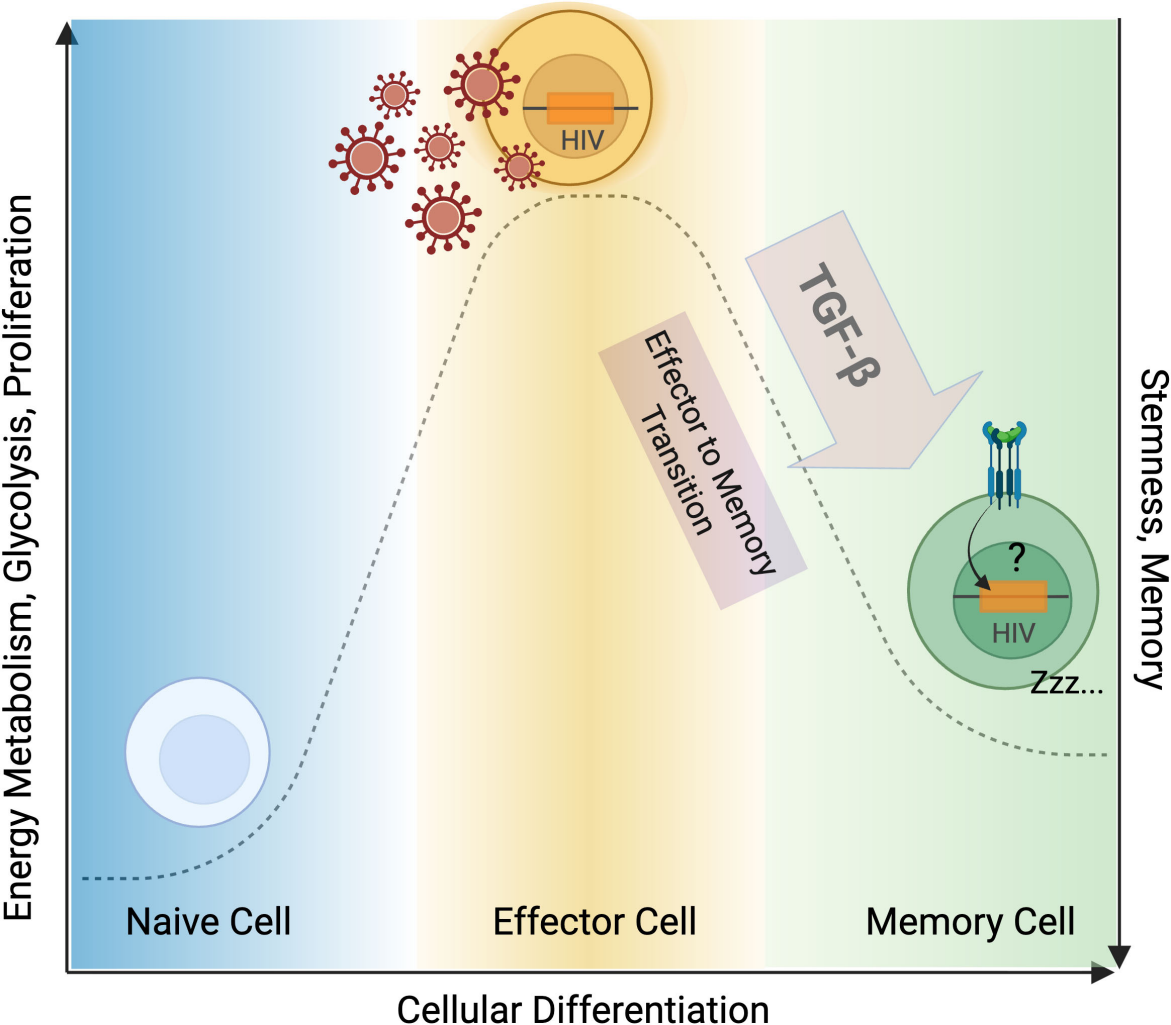
TGF-β contributes to HIV latency. During HIV infection, naïve CD4^+^ T cells become activated and differentiate into effector T cells, which are susceptible to HIV infection. Effector T cells are characterized by increased proliferation, energy metabolism, and glycolysis. TGF-β mediates the transition of HIV-infected effector T cells to a latently infected quiescent memory phenotype. TGF-β maintains infected memory CD4^+^ T cells in a latent state, contributing to HIV persistence of infection. Figure created with BioRender.com.

Other primary cell latency systems have been developed based on similar principles, including the LARA (latency and reversion assay) model (155, 161). This model was based on the observation that the majority of the inducible viral reservoir *in vivo* is present in effector memory (T_em_) subsets rather than central (T_cm_) or transitional (T_tm_) memory CD4^+^ T cells (155) (**Figure 5**). This is in alignment with other studies that have reported an enrichment of the HIV reservoir in T_em_ cells compared to other memory subsets (162, 163). However, several recent studies have suggested that the enrichment of intact proviruses in specific cell subsets may depend on the timing of ART (164, 165), immune responses (166) and sex differences (167). That being said, the association between an effector memory signature and higher reservoir inducibility is also supported by modeling studies (168), cross-sectional studies (169), and evidence suggesting that cellular differentiation promotes viral expression and immune recognition (170, 171).

The LARA model begins with the isolation of T_cm_, T_tm_, and T_em_ CD4^+^ T cell subsets from HIV-negative donors, which are subsequently infected with HIV (161). Culture conditions are designed to mimic the lymph node microenvironment that supports long-term T cell survival and quiescence. Specifically, cells are maintained in media containing TGF-β and IL-7 (cytokines involved in memory T cell establishment and survival (172)) that is further supplemented with conditioned medium from the H-80 stromal cell line, which is rich in TGF-β1, TGF-β2, TGF-β3, and IL-9. Over two weeks, this culture system induces a marked decrease in T_tm_ and T_em_ populations, accompanied by a relative increase in quiescent T_cm_ cells (155, 161). Given that T_em_ cells more readily revert to a virus-producing phenotype (171), this shift toward a more undifferentiated memory state suggests a mechanism by which TGF-β signaling may facilitate HIV latency maintenance. TGF-β signaling is known to promote an undifferentiated, stem-like transcriptional profile in T cells through both epigenetic and metabolic mechanisms (49, 173, 174), though the exact contributions of these pathways to HIV latency remain to be determined (**Figure 5**).

Another line of evidence supporting TGF-β’s role in maintaining HIV latency comes from studies showing that pharmacological inhibition of TGF-β signaling enhances latency reversal across multiple model systems (62, 133). Galunisertib, a small-molecule inhibitor of TGFβRI (ALK5) that reached Phase I/II clinical development, prevents SMAD2/3 phosphorylation and downstream signaling through the canonical pathway (175). *In vitro*, TGF-β prevented PMA-mediated reactivation of viral transcription in both cell line models and in primary CD4^+^ T cells isolated from PBMCs of PWH, but TGF-β-blockade using galunisertib is sufficient to restore reactivation (133). Furthermore, *in vivo*, galunisertib promoted viral reactivation in a small cohort of seven SIVmac251-infected rhesus macaques on long-term ART (63). This observation was subsequently confirmed in a follow-up study of eight SIVmac239M-infected rhesus macaques on ART, where latency reversal was documented in both blood and tissue compartments using classical molecular assays as well as SIV-envelope immuno-PET/CT imaging (62). Mechanistically, TGF-β blockade *in vivo* was associated with transcriptional reprogramming toward a transitional effector phenotype characterized by increased expression of activation- and metabolism-related transcriptional programs (**Figure 5**), yet notably without upregulation of canonical activation or proliferation markers (62). These findings suggest that HIV latency reversal could be achieved through TGF-β blockade without risk of inducing systemic immune activation or inflammation.

Finally, recent clinical studies characterizing the transcriptionally silent, replication-competent reservoir in long-term virologically suppressed individuals by comprehensive single-cell sequencing and phenotypical analysis provide additional evidence for a role of TGF-β in shaping the HIV reservoir (166). These studies revealed enrichment of TGFβRI expression in CD4^+^ T cells harboring intact proviral transcripts, along with markers of T cell exhaustion, such as PD-1 and TIGIT (166). These findings suggest that TGF-β signaling contributes to maintenance of the latent reservoir and promotes the persistence of these cells in PWH on ART, even after decades of suppressive therapy.

Taken together, evidence from *in vitro* latency models, *ex vivo* studies, *in vivo* blockade experiments, and clinical specimen characterization converge to identify TGF-β as a key regulator that links T cell quiescence and viral persistence. TGF-β appears to contribute to HIV latency through at least two mechanisms: first, by facilitating the effector-to-memory cell transition (176) leading to latency establishment through transcriptional and epigenetic remodeling; and second, by maintaining cellular quiescence and stem-like properties through metabolic suppression, particularly via mTOR inhibition (173) and its effects on mitochondria respiration (54). These mechanisms likely act synergistically to both establish and stabilize HIV latency across diverse biological compartments, raising the threshold for latency reactivation and promoting the selection of long-lived reservoir cells that persist despite decades of suppressive ART (**Figure 5**). That being said, significant gaps remain in our understanding of TGF-β’s role across these different reservoir compartments, cell types, and experimental systems, necessitating further investigation to elucidate how TGF-β signaling shapes HIV persistence *in vivo* and how it can be targeted to eliminate the viral reservoir and induce virologic control.

## 6 Conclusions and perspectives

Elevated TGF-β levels in PWH establish this cytokine as a central mediator of HIV pathogenesis and persistence with direct links to latency maintenance, immunosuppression, tissue dysfunction, and accelerated aging. While substantial progress has been made in understanding TGF-β’s multifaceted roles in HIV infection, critical questions remain regarding underlying mechanisms and the potential of TGF-β as a therapeutic target.

*In vivo* blockade of TGF-β in SIV-infected macaques on ART has been shown to enhance antiviral T cell responses (62, 63), demonstrating that pharmacological intervention can partially restore immune function in the setting of chronic infection. However, the magnitude of immune enhancement achieved through TGF-β blockade may not match that observed with PD-1 pathway inhibition (177), which has demonstrated robust restoration of T cell effector functions in the context of chronic HIV infection and cancer (178, 179). Despite this, TGF-β blockade offers unique advantages through its additional effects on myeloid and NK cells cell populations. Notably, the combined enhancement of CD8^+^ T cell, myeloid, and NK cell effector mechanisms positions TGF-β blockade as a promising immune enhancement strategy, especially when used in combination with therapeutic antibodies that can engage NK cells through their Fc receptors to eliminate infected cells.

Beyond immunosuppression, TGF-β drives fibrotic development across multiple tissues in PWH. However, clinical translation of TGF-β blockade for the treatment of fibrotic diseases has proven challenging and has suggested that fibrotic pathways in chronic diseases are sustained by complex and redundant mechanisms. TGF-β may initiate fibrotic remodeling, but perpetuation of fibrosis likely involves multiple parallel pathways—including PDGF signaling, integrin-mediated mechanotransduction, and cross-talk with other pro-fibrotic cytokines such as CTGF and IL-13—that compensate loss of TGF-β signaling (180–182). This redundancy implies that successful therapeutic strategies for HIV-driven fibrosis and accelerated aging may require simultaneous targeting of multiple pathways rather than TGF-β blockade alone. Nevertheless, given TGF-β’s centrality in initiating and maintaining these pathological processes, additional investigations are warranted, focusing on combination strategies that address both TGF-β-dependent and TGF-β-independent mechanisms.

TGF-β’s dual effects on HIV infection make it a truly unique target in curative strategies. TGF-β increases susceptibility to HIV infection by upregulating CCR5 expression on CD4^+^ T cells while also promoting viral latency by suppressing viral transcription and driving infected cells into quiescent, memory-like states. This dual role makes TGF-β blockade particularly appealing as a means to simultaneously promote latency reactivation (enabling immune clearance of reactivated reservoir cells) and limit new infections or reservoir reseeding during treatment interruption.

Moreover, two additional, often-overlooked aspects of TGF-β function may be relevant to understanding its role in HIV persistence on ART. First, TGF-β signaling is known to limit IL-7-mediated homeostatic proliferation of memory CD4^+^ T cells (183). this raises the possibility that TGF-β blockade may inadvertently contribute to the expansion of T cell clones carrying HIV proviruses. Clonal expansion of reservoir cells is a major contributor to reservoir maintenance in PWH on ART (184, 185). Hence, this potential off target effect of TGF-β blockade cannot be quickly discounted. Second, TGF-β signaling is essential for maintaining tissue residency programs in T cells, including expression of retention signals that keep tissue-resident memory T cells (T_rm_) localized within peripheral tissues (137, 186). Blocking TGF-β may disrupt these tissue residency signals, leading to mobilization and recirculation of latently infected T_rm_ cells from tissue compartments. This redistribution could facilitate immune-mediated elimination of reservoir cells by exposing them to systemic immune surveillance and by making them more accessible to therapeutic interventions such as checkpoint inhibitors, bNAbs, or cytotoxic T lymphocytes. Neither of these aspects has been addressed in current *in vivo* or *ex vivo* studies, yet both deserve attention as they could substantially influence the net impact of TGF-β blockade on reservoir dynamics.

Given these multifaceted roles in HIV infection and immune regulation, the timing and method of TGF-β blockade are likely to be critical determinants of therapeutic success. Current published *in vivo* studies have employed galunisertib, a small-molecule inhibitor of TGFβRI that offers reversible and titratable inhibition, as a means to establish TGF-β blockade. Intermittent dosing regimens in humans and in SIV-infected macaques have proven extremely safe, without inducing compensatory inflammatory cytokine release or systemic immune activation (62, 187). In contrast, alternative approaches such as irreversible blockade of TGF-β or its receptors using monoclonal antibodies may trigger compensatory upregulation of parallel inflammatory pathways_—_potentially blunting therapeutic benefit or introducing unacceptable toxicity. Equally critical is the timing of TGF-β blockade relative to disease course and treatment status. Administering TGF-β inhibitors during analytical treatment interruption (ATI) may drive latency reversal and accelerate viral rebound kinetics before immune responses can be sufficiently enhanced to control replication. Hence, this approach could paradoxically worsen outcomes during ATI. In support of this, a recent analysis of rebound kinetics in SHIV-infected infant rhesus macaques treated with ART early (day 5–7 post-infection) found that enhanced TGF-β signaling in pre-ATI CD4^+^ T cells, rather than its blockade, was associated with post-ATI virologic control (188). In contrast, initiating TGF-β blockade at the time of ART initiation—when infected cells are naturally transitioning from activated effector states to quiescent memory phenotypes—may represent a more strategic intervention. TGF-β signaling facilitates the effector-to-memory transition (176, 189), and blocking this pathway during early ART could disrupt entrance into latency, thereby preventing or limiting reservoir establishment.

Beyond direct therapeutic implications, *in vivo* studies of TGF-β blockade may provide mechanistic insights into the epigenetic and metabolic underpinnings of HIV latency and immune dysfunction. TGF-β signaling regulates chromatin accessibility, histone modifications, and metabolic pathways that maintain T cell quiescence and stem-like properties. By observing how TGF-β blockade alters viral reactivation, immune phenotypes, and transcriptional programs *in vivo*, we can identify key regulatory nodes that control metabolic and epigenetic regulation of latency maintenance and contribute to effective immune responses. Thus, while TGF-β blockade may or may not prove to be the ultimate therapeutic solution, the knowledge gained from understanding the role of TGF-β in HIV persistence may be invaluable for rational design of HIV cure strategies.

Despite these advances, several critical questions remain unanswered. First, TGF-β’s variable efficacy in blocking latency reversal across different models and with different LRAs suggests model- and stimulus-specific mechanisms that remain poorly understood. Second, the molecular mechanisms underlying TGF-β-mediated latency establishment in primary CD4^+^ T cells remain largely uncharacterized. Third, while early work in the U1 cell line suggests TGF-β maintains myeloid latency, the mechanisms involved, and their physiological relevance, remain unexplored. Studies in primary monocyte-derived and tissue-resident macrophages are needed to determine whether TGF-β enforces myeloid latency through mechanisms similar to those in T cells or via distinct pathways. Finally, and perhaps most critically, the role of TGF-β in maintaining latency within tissue reservoirs–where the majority of the HIV reservoir resides–remains poorly defined. It is unclear whether TGF-β’s latency-maintaining effects are universal across tissue sites or vary in a tissue-, cell type-, or anatomical compartment-specific manner. TGF-β acts in concert with other factors that modulate its downstream effects. Tissue microenvironments contain complex cytokine milieus including IL-7, IL-10, IL-15, and others that may synergize with, antagonize, or modulate TGF-β’s effects on latency. Understanding these context-dependent interactions will be crucial for determining whether and how TGF-β signaling can be therapeutically targeted to disrupt the HIV reservoir without compromising its essential physiological functions in immune regulation and tissue homeostasis.

Defining these mechanisms will be pivotal to determine whether targeted modulation of TGF-β can be safely employed to alleviate fibrosis, attenuate accelerated aging-related comorbidities, enhance immune control of HIV, and synergize with emerging curative strategies—including bNAbs, therapeutic vaccines, and checkpoint inhibitors—to achieve a functional cure. The path forward requires not only continued investigation of TGF-β as a direct therapeutic target, but also deeper exploration of the biological pathways it regulates with the ultimate goal of developing comprehensive, multi-pronged interventions that address the interconnected challenges of immune dysfunction, tissue damage, and viral persistence in PWH.
